# Radiation-free interlocking intramedullary nailing of three-hundred and seventy long bone fractures in Ogbomoso, Nigeria

**DOI:** 10.1038/s41598-021-89544-2

**Published:** 2021-05-12

**Authors:** Stephen Adesope Adesina, Samuel Uwale Eyesan, Innocent Chiedu Ikem, Olalekan Akeem Anipole, Isaac Olusayo Amole, Akinsola Idowu Akinwumi, Philip Oluyemi Bamigboye, Adewumi Ojeniyi Durodola

**Affiliations:** 1 Department of Family Medicine, Bowen University Teaching Hospital, Ogbomoso, Oyo State Nigeria; 2 Department of Family Medicine, Bowen University, Iwo, Osun State Nigeria; 3 Department Surgery, Bowen University Teaching Hospital, Ogbomoso, Oyo State Nigeria; 4 Department Surgery, Bowen University, Iwo, Osun State Nigeria; 5 Department of Orthopaedic Surgery and Traumatology, College of Health Sciences, Obafemi Awolowo University, Ile-Ife, Osun State Nigeria; 6 Department of Family Medicine, Afe Babalola University, Ado-Ekiti, Ekiti State Nigeria

**Keywords:** Health care, Medical research

## Abstract

Long bone fracture care in developing countries remains largely different from that of the developed world where closed reduction and internal fixation with locked intramedullary nail is the standard treatment. This study in a developing country presents the pattern and outcome of treatment of 370 long bone fractures using the SIGN nail over a five-year period in order to underline the wide array of patients and fractures treatable with the nail. Using a prospective descriptive approach, all the 342 patients with 370 fractures of the humerus, femur and tibia treated from July 2014 to June 2019 were studied. The fractures were reduced without image intensifier or fracture table and fixed with the SIGN nail. Post-discharge, the patients were followed up at the out-patient clinic. The mean age of the patients was 43.45 years with a range of 10–99 years. Sixty-six percent were males who were mostly injured in motorcycle accidents. Femur, tibia and humerus fractures accounted for 59.7%, 28.4% and 11.9% respectively. Eighty-six percent were diaphyseal fractures, 73% were fresh and the main previous treatment was traditional bone setting. Deep infection occurred in 4.9%, 66.0% achieved knee flexion > 90° by sixth week, the majority achieved full weight bearing and could squat and smile by 12th week. The SIGN nail is versatile, useful for treating a wide range of fractures in most age groups particularly in developing countries where orthopaedic fractures are prevalent but the more sophisticated facilities are lacking or poorly maintained.

## Introduction

In the developed world, the standard treatment of long bone diaphyseal fractures is closed reduction or limited open reduction and fixation with locked intramedullary nail done with the aid of image intensifiers, power reaming and fracture tables^1^. Such ideal fracture care with modern implants is often lacking in the developing countries, where, ironically, the majority of these injuries occur owing to poor roads and precarious transport systems. Fracture victims in such countries are thus unfortunately committed to non-operative treatment with cumbersome poverty-propagating prolonged traction and cast splinting or complications-laden surgical fixation with less effective and outdated implants^2^. Ominously, while trauma is the fastest growing epidemics worldwide currently, road traffic injuries alone are predicted to become the third largest contributor to the global burden of disease by 2030, and most of these in the developing countries^3^.

Ogbomoso, where Bowen University Teaching Hospital is located is a semi-urban city in South-Western Nigeria made up largely of artisans, poorly-remunerated civil servants, subsistence farmers and small business owners. The hospital serves other nearby towns composed of similar populations. Until July 2014, the mainstay of treatment of diaphyseal fracture of the femur was open reduction with Küntscher nails while femoral metaphyseal fractures as well as fractures of the tibia and humerus were largely treated with casts with varying degree of success. However, this story changed in early 2014 when the Surgical Implant Generation Network (SIGN) (Richland, WA, USA) reached the hospital with the SIGN intramedullary (IM) nailing system, a locked IM nailing system which can be done without image intensifier, fracture table or power reaming^4^. The SIGN IM nailing has subsequently made most of the long bone fractures seen in our hospital to become treatable surgically. We present in this study the characteristics of 342 patients as well as the pattern and outcome of treatment of their 370 long bone fractures using the SIGN IM nailing implants over a period of 60 months, with a view to underline the wide array of patients and fractures treatable with this versatile nail.

## Materials and methods

This study is a prospective descriptive study of 342 patients with 370 fractures of the humerus, femur and tibia who were treated in our centre from July 2014 to June 2019. The Gustilo-Anderson system was used to classify open fractures. Each patient’s fracture(s) was/were reduced open or closed without image intensifier or fracture table. The bone canals were manually reamed and the fractures were fixed with the SIGN IM locked nail with not less than two screws. The surgical procedure was as described by the manufacturer^5^. All the patients had ceftriaxone or other antibiotics administered for four to five days or longer depending on the fracture type or concomitant injuries. Pre- and post-operative anteroposterior and lateral radiographs were taken for all patients. The patients were ambulated with or without aid from the first post-operative day, as their conditions permitted. They were discharged from the hospital starting from post-operative day four onwards as considered expedient by the health care team and their care givers.

The location and morphology of the fractures were defined according to the Arbeitsgemeinschaft für Osteosynthesefragen/Orthopaedic Trauma Association (AO/OTA) guideline for classification of long-bone fractures^6^: the locations were classified as proximal end segment, diaphyseal segment, distal end segment or combinations of these (Table 1) while morphology is stated using the alphanumeric codes for each of humerus, femur and tibia (Table 2). New fractures were defined as fractures that were presented to our hospital within three weeks of occurrence and without any prior failed definitive treatment whereas fractures that were presented to our hospital beyond three weeks post-occurrence or after a failed definitive treatment were labelled old fractures (also included non-unions, mal-unions and delayed unions)^7^.

**Table 1:** Patients’ and fracture characteristics.

Total Variable		n (%)
Age group (years) (n = 342) *Mean age: 43.45 years* *Age range: 10–99 years*	10–19	19 (5.6)
	20–29	65 (19.0)
	30–39	79 (23.1)
	40–49	65 (19.0)
	50–59	41 (12.0)
	60–69	28 (8.2)
	70–79	32 (9.3)
	80–89	9 (2.6)
	90–99	4 (1.2)
Gender (n = 342)	Male	227 (66.4)
	Female	115 (33.6)
Cause of fracture (n = 342)	Assault	7 (2.0)
	Fall	62 (18.2)
	Motor vehicle accident	77 (22.5)
	Motorcycle accident	140 (40.9)
	Pedestrian injury	56 (16.4)
Co-morbidity (n = 342)	No co-morbidity or controlled Hypertension	295 (86.3)
	Co-morbidity that may delay wound healing(DM, HIV, Pregnancy, SCD)	23 (6.7)
	Co-morbidity that may impair ambulation (OA, Obesity, Osteoporosis)	22 (6.4)
	Visual/hearing impairment	2 (0.6)
Death while on Admission (n = 342)	Yes	3 (0.9)
	No	339 (99.1)
Fractured bone (n = 370)	Humerus	44 (11.9)
	Femur	221 (59.7)
	Tibia	105 (28.4)
Fracture Side (n = 370)	Right	174 (47.0)
	Left	196 (53.0)
Fracture type (n = 370)	Closed	304 (82.2)
	Gustilo I	20 (5.4)
	Gustilo II	11 (2.9)
	Gustilo IIIA	25 (6.8)
	Gustilo IIIB	10 (2.7)
Fracture location (n = 370)	Proximal end segment	27 (7.3)
	Diaphyseal segment	320 (86.5)
	Distal end segment	23 (6.2)
Duration of fracture (n = 370)	Fresh fractures	270 (73.0)
	Old fractures	100 (27.0)
Initial definitive fracture treatment modality (n = 370)	No previous definitive treatment	267 (72.2)
	Cast	14 (3.8)
	Traditional bone setting	74 (20.0)
	External Fixator	3 (0.8)
	Traction	2 (0.5)
	ORIF (plate and screw, IM nail)	10 (2.7)

*DM* diabetes mellitus, *HIV* human immunodeficiency virus infection, *SCD* Sickle cell disease, *OA* Osteoarthritis, *ORIF* open reduction and internal fixation.

**Table 2:** Fracture morphology (OA/OTA Classification).

	OA/OTA Classification	n (%)
Humerus fractures (n = 44)	12-A1	2 (4.5)
	12-A2	11 (25.0)
	12-A3	13 (29.5)
	12-B2	9 (20.5)
	12-B3	5 (11.4)
	12-C3	3 (6.8)
	13-A3	1 (2.3)
Femur fractures (n = 221)	31-A1	4 (1.8)
	31-A2	1 (0.5)
	31-A3	21 (9.5)
	32-A1	15 (6.8)
	32-A2	20 (9.0)
	32-A3	47 (21.3)
	32-B2	50 (22.6)
	32-B3	23 (10.4)
	32-C2	4 (1.8)
	32-C3	17 (7.7)
	33-A2	7 (3.1)
	33-A3	3 (1.4)
	33-C1	2 (0.9)
	33-C2	3 (1.4)
	33-C3	4 (1.8)
Tibia fractures (n = 105)	41-A2	1 (0.9)
	42-A1	10 (9.5)
	42-A2	9 (8.8)
	42-A3	28 (26.7)
	42-B2	18 (17.1)
	42-B3	16 (15.2)
	42-C2	8 (7.6)
	42-C3	12 (11.4)
	43-A2	2 (1.9)
	43-A3	1 (0.9)

Data on patient and fracture characteristics were collected prospectively and entered into the lead author’s computer post-operatively and at follow-ups. The patients were followed up with plain radiographs and test of ability to squat and smile (for femur and tibia fractures only) or shoulder abduction and external rotation (for humerus fractures only). The follow-ups were done at least twice—at six weeks and 12 weeks—but also at six and 12 months if ambulation or fracture healing was not achieved at 12th week follow-up. The time taken to achieve full weight bearing and knee flexion/shoulder abduction beyond ninety degrees, as well as the presence of infection was also noted.

All ethical principles guiding a research of this nature were duly adhered to while undertaking the study. All patients aged 18 years and above (or the parents of those younger than 18 years) gave informed consent to be included in the study. The study was approved by the Institutional Review Board of Bowen University Teaching Hospital, Ogbomoso, Nigeria. The data were analysed with SPSS version 23 (IBM Corp, New York, USA).

## Results

Table 1 shows that the percentage of patients within age groups 30–39 was the highest (23.1%) followed closely by age groups 20–29 years (19.0%) and 40–49 years (19.0%), with a mean age of 43.45 years. Male patients constituted about two-thirds (66.4%) of the population. The highest proportion (40.9%) of the patients sustained their fractures in motorcycle accident. Most (86.3%) of the patients either had no co-morbid conditions or an easily controlled hypertension. Three patients died while on admission. The largest proportions of the fractures involved the femur (59.7%), affected the left limb (53.0%) and were closed (82.2%). There were more fractures located in the diaphyseal segment (86.5%) than other sites but it is noteworthy that a good number of fractures in the proximal and distal end segments were treated. The majority of the fractures were fresh (73.0%). Although most (72.2%) of the fractures received no initial definitive treatment before SIGN nail was inserted, traditional bone setting was the most frequent (20.0%) treatment method among those that were treated.

Table 2 depicts the OA/OTA classification of the fractures. Most of the humerus fractures were simple oblique (12-A2; 25.0%), simple transverse (12-A3; 29.5%) and intact wedge (12-B2; 20.5%) fractures of the diaphysis. The proportion of the femur fractures that were simple transverse (32-A3) and intact wedge (32-B2) diaphyseal fractures were highest (21.3% and 22.6% respectively). However, also having fairly high proportions were fragmentary wedge diaphyseal fractures (32-B3; 10.4%), simple oblique diaphyseal fractures (32-A2; 9.0%), intertrochanteric (reverse oblique) fractures (31-A3; 9.5%), multifragmentary fragmentary segmental fractures (32-C3; 7.7%) and simple spiral diaphyseal fractures (32-A1; 6.8%). The highest proportion of the tibia fractures were simple transverse diaphyseal fractures (42-A3; 26.7%).

**Figure 1:**
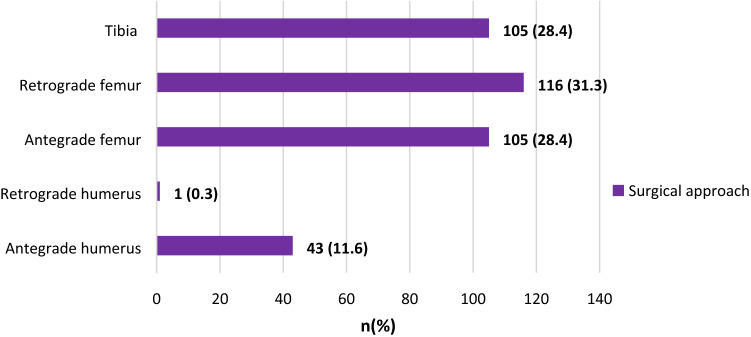
Surgical approach.

**Figure 2:**
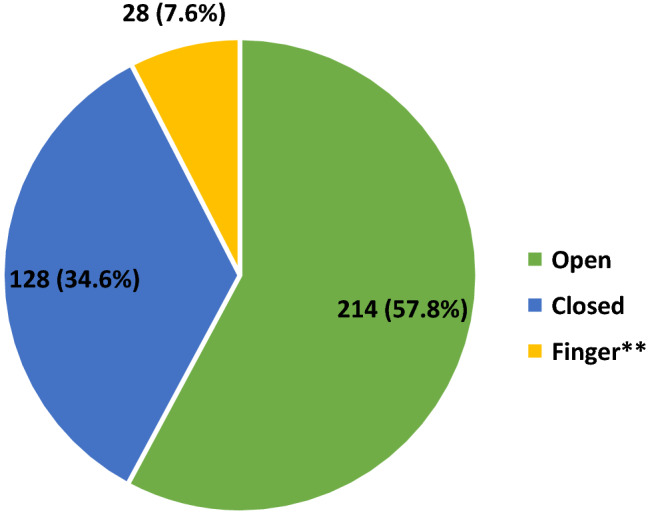
Fracture reduction method. ** "Finger reduction" = reduction achieved with one finger of the surgeon dipped into the fracture site via a mini incision of ≤3cm.

In Fig. 1, it is observed that more of the femur fractures were operated using the retrograde (31.4%) than antegrade approach. It is also noteworthy that one of the humerus fractures was fixed via a retrograde approach. Figure 2 shows that more than one-third (34.6%) of the fractures were reduced closed. This is remarkable considering the fact that the surgeries were done without intra-operative imaging.

In Table 3, it can be observed that well above one-half (58.4%) of the fractures were operated within one week of occurrence, 27 (7.3%) of the fractures were treated with an accompany side plate to the SIGN nail and the majority of the fracture cases were discharged home within the first post-operative week. Almost two-thirds (66.0%) of the fracture cases achieved knee flexion (femur and tibia fractures only) or shoulder abduction (humerus fractures only) beyond ninety degrees at the 6-week follow up visit. The majority of the cases had achieved full weight bearing and could squat and smile (for femur and tibia fractures only) or do shoulder abduction and external rotation (for humerus fractures only) by 12-week follow up. In more than one-half of the fractures (58.4%), there was radiographic evidence of healing in the 6-week follow up visit and most of the fractures healed without infection.

**Table 3:** Treatment details and outcomes.

Total Variable (n = 370)		n (%)
Time between occurrence of fracture and surgery	First week	216 (58.4)
	Second week	29 (7.8)
	Third week	10 (2.7)
	Fourth week	10 (2.7)
	After fourth week	105 (28.4)
Side plate used?	No	343 (92.7)
	Yes	27 (7.3)
Duration of Admission	Died on admission	3 (0.8)
	Discharged first post-op week	310 (83.8)
	Discharged second post-op week	37 (10.0)
	Discharged third post-op week	5 (1.4)
	Discharged fourth post-op week or after	15 (4.0)
Knee flexion/Shoulder abduction > 90 present at:	6week follow up	244 (66.0)
	12-week follow up	71 (19.2)
	6-month follow up	19 (5.1)
	After 6-month follow up	6 (1.6)
	Not achieved	13 (3.5)
	Absent (stiff before surgery)	8 (2.2)
	Absent follow up	9 (2.4)
Full weight-bearing noted at:	6 week follow up	152 (41.1)
	12-week follow up	172 (46.5)
	6-month follow up	35 (9.5)
	After 6-month follow up	2 (0.5)
	Absent follow up	9 (2.4)
Able to squat and smile at:	6-week follow up	112 (30.3)
	12-week follow up	160 (43.2)
	6-month follow up	50 (13.5)
	after 6-month follow up	11 (3.0)
	Not achieved	20 (5.4)
	Absent (stiff before surgery)	8 (2.2)
	Absent follow up	9 (2.4)
Evidence of healing noted on plain radiograph at:	6-week follow up	216 (58.4)
	12-week follow up	130 (35.1)
	6-month follow up	10 (2.7)
	After 6-month follow up	1 (0.2)
	Not achieved	2 (0.5)
	After repeat surgery	2 (0.5)
	Absent follow up	9 (2.4)
Infection type	None	347 (93.8)
	Superficial	5 (1.3)
	Deep	18 (4.9)

## Discussion

This study has presented the use of the SIGN IM nail for the treatment of 370 fractures of the humerus, femur and tibia in 342 patients whose ages ranged between ten and 99 years (Table 1), underscoring the adaptability of the nail to fracture care in different age groups, the extremes inclusive. This finding offers hope for surgical treatment of children’s and adolescents’ fractures in low and middle income countries where the elastic stable intramedullary nailing which is the current gold standard for surgical fixation of their fractures is often lacking^8,9^. Similarly, the nail is useful in treating elderly patients who may be at risk for non-union (owing to poorer bone and fracture biology or co-morbid medical conditions) if they are treated non-operatively or with other implants^10^.

Of note in this study is the finding that patients within age groups 20–29, 30–39 and 40–49 years (mean age: 43.45 years) accounted for the majority of those treated, and that almost two-thirds (66.4%) were males who mostly sustained their fractures in road traffic accidents as motor vehicle occupants (22.5%), rider/passenger on motorcycles (40.9%) or pedestrians hit by a vehicle (16.4%) (Table 1). While these findings are in consonance with those of previous studies in developing countries^4,11,12^, our finding that the highest proportion (40.9%) of the patients sustained their fractures in motorcycle accident is of particular concern in a country with a poorly developed trauma system. Popularly referred to as *Okada*, commercial motorcycle became the major means of public transportation in many Nigerian towns and cities as a combined effect of economic downturn, rapid urbanization, unemployment and inadequate intra-city public transportation^13,14^. Unfortunately, this has also led to a rise in the number of orthopaedic injuries.

Most (86.3%) of the patients either had no co-morbid conditions or an easily controlled hypertension. This finding appear to reflect the general youthfulness of the study population, as against a majority elderly population in which a higher occurrence of co-morbidity would be expected. The three patients who died on admission included (i) a 66-year old male hypertensive with AO/OTA 32C3 fracture operated 8 days post injury who died less than 24 h post-operatively; (ii) a 46-year old male with AO/OTA 42C3 fracture operated 4 days post injury, who also had contralateral knee dislocation and died post-operative day 6 while training to ambulate; (iii) a 70-year old female hypertensive with AO/OTA 32-A1 fracture operated 3 days post injury who died less than 24 h post-operatively. As previously documented^15,16^, relations declined autopsy but the cause of death in all of them appeared to be from deep venous thrombosis/pulmonary thromboembolism.

Similar to what has been found in previous studies^4,17^, we also found that femur fractures (59.7%) accounted for the largest proportion of the fractures treated, followed by tibia (28.4%) fractures. However, the number of humerus fractures (11.9%) treated was higher than has been previously found in other studies^4,17^, although a number of other studies described the use of the SIGN nail in femur and tibia fractures only^11,12,18^. It is notable that 100 (27.0%) of the fractures were old fractures which included non-unions, mal-unions, delayed unions and fractures that were presented to our hospital beyond three weeks post-occurrence or after a failed definitive treatment (Table 1). Out of these, a whopping 74 were initially treated by traditional bone setters. These findings underscore the versatility of the SIGN nail in treating complications resulting from fracture treatment by other modalities. This is especially important in our setting where traditional bone setting and the attendant complications are pervasive^19^.

**Figure 3:**
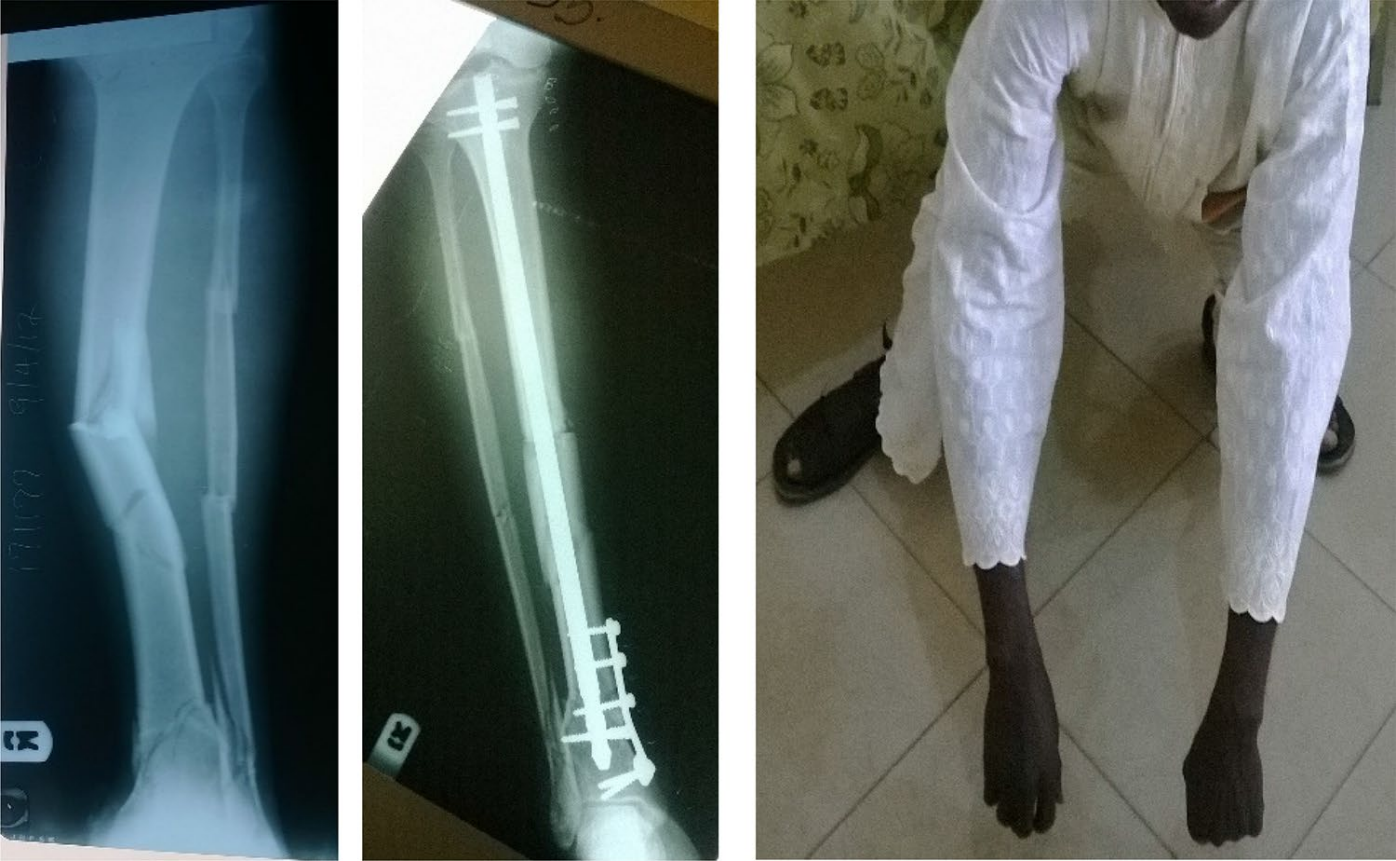
Pre- and post-operative radiographs and “squat and smile” photograph (6-month follow-up) of a 50-year old man who had AO/OTA 32C3 (not shown) and 42C3 fractures. The SIGN nail was combined with a side plate to treat the most distal tibia fracture.

**Figure 4:**
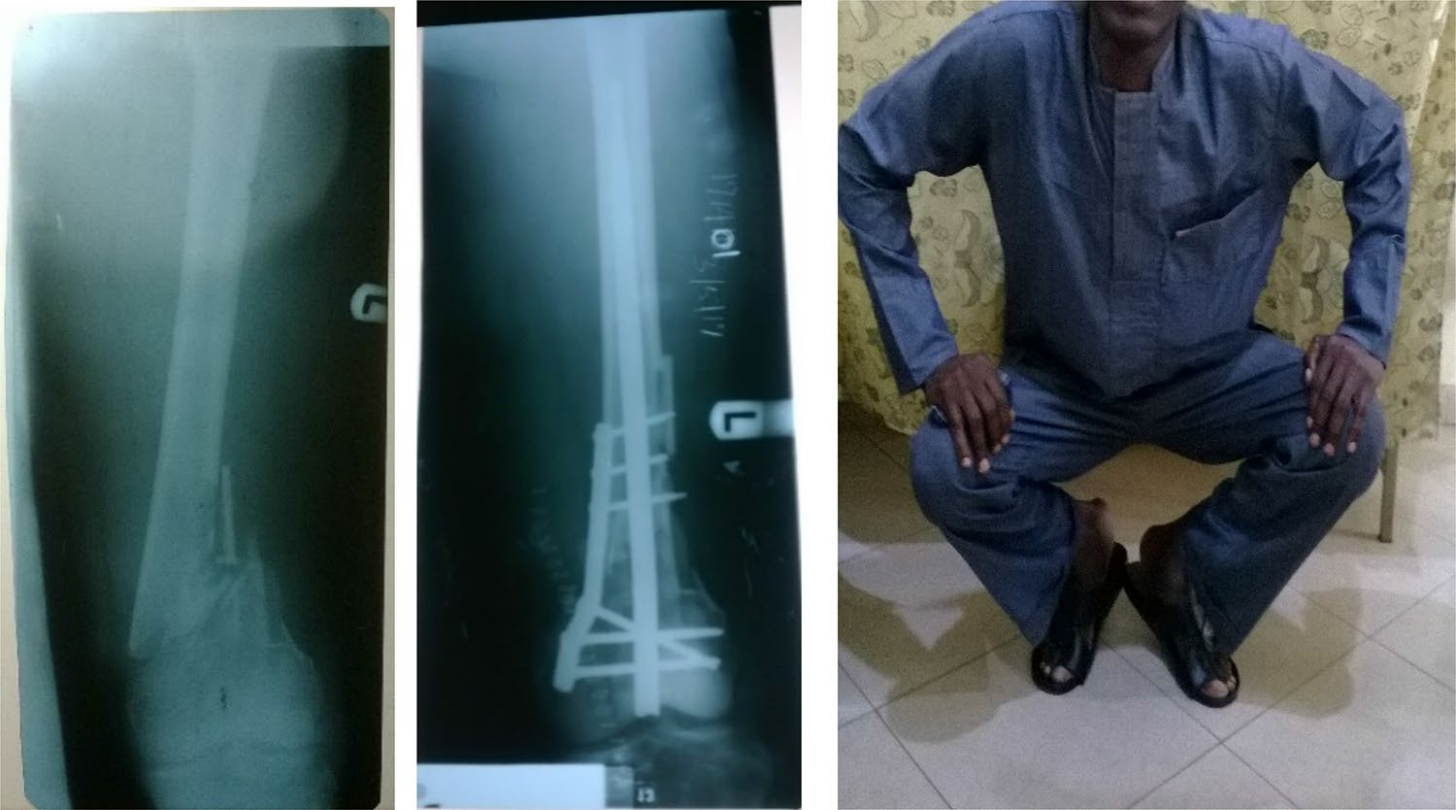
Pre- and post-operative radiographs and “squat and smile” photograph (6-month follow-up) of a 43-year old man in whom the SIGN fin nail was used with a narrow direct compression plate (DCP) to treat a comminuted metaphyseal femur fracture.

**Figure 5:**
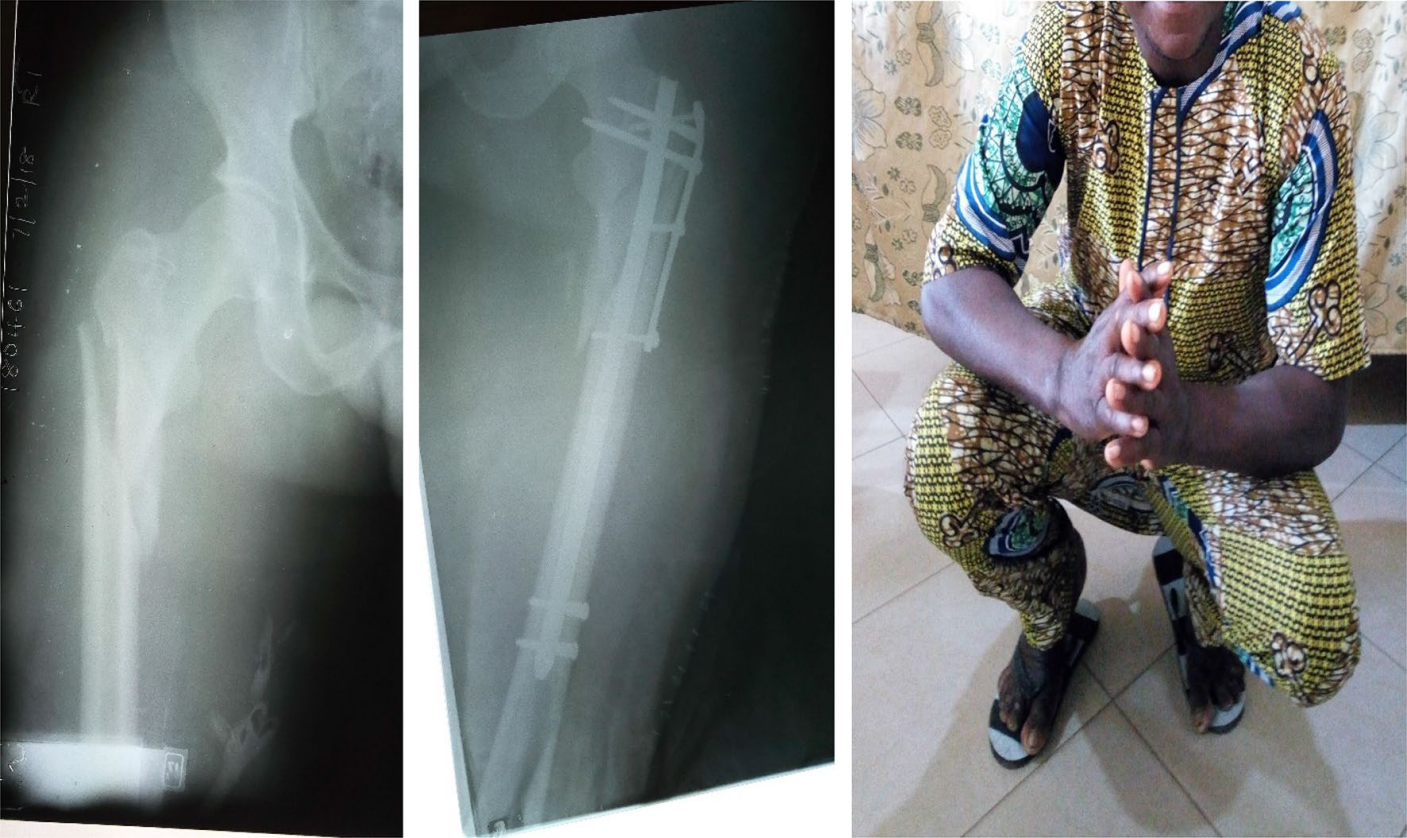
Pre- and post-operative radiographs and “squat and smile” photograph (6-week follow-up) of a 33-year old man who had the SIGN nail combined with the SIGN HV plate to treat a reverse oblique femur fracture with diaphyseal extension.

Whereas most of the fractures (86.5%) were in the diaphyseal segment (as defined in the 2018 AO/OTA Fracture and Dislocation Classification Compendium)^6^, it is worth mentioning that 50 (13.5%) of the fractures were in the proximal and distal end segments (Table 1). We were able to achieve stable fixation and satisfactory outcome in these end segment fractures by combining the SIGN IM nail with side plates provided by SIGN Fracture Care International (Figs. 3, 4 and 5). All but one of the humerus fractures treated were in the diaphysis and they were mostly simple (12-A) or intact wedge (12-B2) fractures (Table 2). The femur and tibia fracture patterns were similarly majority diaphyseal fractures but also having fairly high proportions among femur fractures were 31-A3 (intertrochanteric [reverse oblique]) fractures and 32-C3 (multifragmentary fragmentary segmental fractures) (Table 2).

Although the surgeries were done without intra-operative imaging, we aimed to achieve close reduction or finger reduction of the fresh fractures, and we achieved this aim in 156 cases which is 57.8% of the 270 fresh fractures (Table 1 and Fig. 2). This differs from a number of earlier studies in developing countries where reduction is mostly open in the absence of image intensifier^11,12,18,20^. The factors which from our observation facilitated this included: operating the fractures within 72 h of occurrence; using the retrograde approach for mid-shaft and distal femur fractures; using the Alaska Surgical Support Triangle for femur and tibia fractures. Hence, well above one-half (58.4%) of the fractures were operated within one week of occurrence and, contrary to what is reported by some previous researchers^12,17,18^, more of the femur fractures were operated using the retrograde (116) than antegrade (105) approach. Nevertheless, we should mention that close reduction was achieved for some of the femur fractures fixed using the antegrade approach, too.

The outcome measures in our study included knee flexion (femur and tibia fractures only) or shoulder abduction (humerus fractures only) beyond ninety degrees, full weight bearing, ability to squat and smile (for femur and tibia fractures only) or do shoulder abduction and external rotation (AER) (for humerus fractures only), radiographic evidence of healing, and occurrence of infection (Table 3). The nine cases (2.4%) for which there was no follow-up included the three patients who died while on admission. The 3.5% knee stiffness (flexion less than 90°) rate, is less than what some earlier studies reported^11,12,18,20^.

The majority of the cases had achieved full weight bearing and could squat and smile or do shoulder abduction and external rotation by 12-week follow up. There was radiographic evidence of healing in 216 (58.4%) of the fractures by the 6-week follow up visit and this rose to 346 (93.8%) by the 12-week follow up. Two patients had an exchange nailing before their fractures healed. These findings indicate the SIGN nail achieves a stable fixation that allows early mobilization and faster healing that is comparable to those of the developed world where modern sophisticated modalities of fracture care are employed. While 347 (93.8%) fractures healed without infection, 5(1.3%) surgical sites had superficial infection which healed with antibiotic treatment. Another 18 (4.9%) cases had deep infection on account of which, following the healing of the fractures, the implants were removed. We however observed that all of the cases in which there was deep infection were open fractures.

In conclusion, from the pattern of the 370 fractures of our 342 patients and the outcome of their treatment, this study has shown the SIGN IM nail to be a very adaptable one that is useful for treating a wide range of fractures, whether old or fresh, in most age groups. Furthermore, the fact that its use is not necessarily dependent on intra-operative radiography, power reaming, and stabilization with a fracture table makes it a particularly invaluable implant in most developing countries where orthopaedic fractures are prevalent but these sophisticated facilities are either absent or poorly maintained.
